# Supplementary material for: Morusin Protected Ruminal Epithelial Cells against Lipopolysaccharide-Induced Inflammation through Inhibiting EGFR-AKT/NF-κB Signaling and Improving Barrier Functions
Source: Int J Mol Sci. 2022 Nov 20;23(22):14428. doi: 10.3390/ijms232214428 (PMC9695078; doi:10.3390/ijms232214428)
Supplement: Supplementary file 1 [file ijms-23-14428-s001.zip › ijms-1927109-supplementary.pdf]

International Journal of
Molecular Sciences

MDPI

Supplementary Materials

Morusin Protected Ruminal Epithelial Cells against Lipopolysaccharide-Induced Inflammation through Inhibiting EGFR-AKT/NF-κB Signaling and Improving Barrier Functions

Chunlei Yang [1,†], Xiangfei Deng [1,†], Linjun Wu [1], Tianrui Jiang [1], Zhengwei Fu [1] and Jinjun Li [2,*]

[1] College of Biotechnology and Bioengineering, Zhejiang University of Technology, Hangzhou 310014, China
[2] Institute of Food Sciences, Zhejiang Academy of Agricultural Sciences, Hangzhou 310021, China
* Correspondence: lijinjun@zaas.ac.cn (J.L.)
† These authors contributed equally to this work.

Table S1. The qPCR primers used in this study.

Gene	NCBI accession	Primer sequence 5′- 3′
TNF-α	NM_001024860	F: GTCCAACTTTAAACAGCTGCACTTA
		R: CCCAAACTTGTGGACCCGA
IL-6	NM_001009392	F: ACACTGACATGCTGGAGAAGATGC
		R: CCGAATAGCTCTCAGGCTGAACTG
NF-κB	XM_027960471	F: ATTGAGCGTCCTGTAACCGTGTTC
		R: TGCACCTCCTCCTTGTCTTCTACC
IL-1β	NM_001009465	F: GCAACCGTACCTGAACCCAT
		R: TGTTGGGTGCAGCTCTTCAT
CCL20	XM_012147649	F: GTCTCCGATACACAGAACGAATACT
		R: TTCACCCACTTCTTCTTTGGATCTG
CD40	XM_012189293	F: AAACAGAATGCCAGTCCTGCGGTAA
		R: ACGCAAGTGGTGTCTGTATTCAAGG
YWHAZ	NM_001267887	F: GATGAAGCCATTGCTGAACTTGA
		R: CAGCTTCGTCTCCTTGGGTA
GAPDH	NM_001190390	F: GTCTTCACTACCATGGAGAAGG
		R: TCATGGATGACCTTGGCCAG

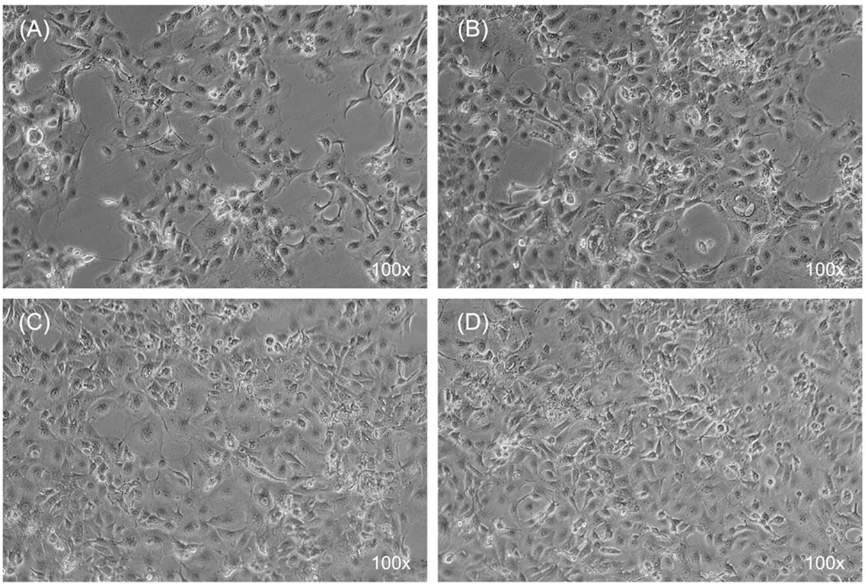

Figure S1. Light microscopy images of the ruminal epithelial cells (RECs) used in this study. **(A)** RECs reached approximately 30% confluency. **(B)** RECs reached approximately 50% confluency. **(C)** RECs reached 70-80% confluency. **(D)** RECs reached 100% confluency.

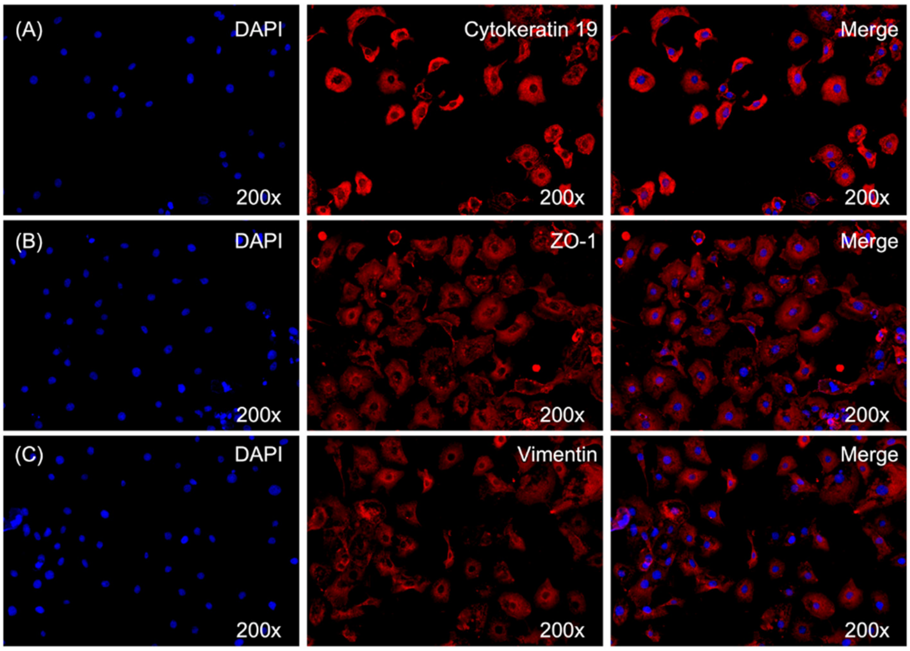

Figure S2. Immunofluorescence staining of the ruminal epithelial cells (RECs) used in this study. **(A)** Fluorescent image showing stained cytokeratin 19. **(B)** Fluorescent image showing stained ZO-1. **(C)** Fluorescent image showing stained vimentin. The antibodies used were anti-Cytokeratin 19 (1:200, Servicebio, GB11197, Wuhan, China), anti-ZO-1 (1:500, Servicebio, GB111402, Wuhan, China) and anti-Vimentin (1:1000, Servicebio, GB111308, Wuhan, China), respectively. DAPI: nuclei were stained with DAPI.
